# Beyond Words: Addressing Ethical Issues in Clinical Documentation through Medical Education

**DOI:** 10.1002/hast.70034

**Published:** 2026-07-20

**Authors:** Danielle Wilfand, Lindsey Grubbs

**Keywords:** medical documentation, stigma, empathy, medical education, structural competency, clinical ethics

## Abstract

*Stigmatizing language in medical documentation is a barrier to achieving patient‐centered care and health justice: it can contribute to patient distrust and alienation, and it appears more frequently in the charts of patients from marginalized groups, especially Black patients, impacting the future care they receive. One powerful avenue to address this issue is within undergraduate medical education; however, while some have investigated educational interventions to improve accuracy and technical proficiency in charting, there is significantly less focus on instruction surrounding the ethical and social consequences of documentation. We contend that incorporating activities designed to elicit critical thinking about language, power, and structural injustice can encourage students to reflect upon and improve their documentation practices and can arm them with a critical lens through which to view the documentation generated by their peers and preceptors as they progress through training and establish the habits that will persist throughout their careers*.

## Essay

Medical charts are complex documents with many audiences and roles: they serve as legal documents, justifications for medical decision‐making, and sources of patient education.^1^ Perhaps the most overlooked function of medical charting is constructing and legitimizing illness experiences and transmitting medical narratives within the health care system. As charts have become increasingly accessible with the adoption of electronic health records (EHRs) and open documentation legislation, their ability to alter the clinical relationship has also expanded.^2^ Research has emphasized that medical charts can alienate patients from the health care system, with one study noting that one in ten patients felt offended or judged by what was written in their medical documentation.^3^ These patient experiences are validated by the literature, which has chronicled common patterns of negative documentation, including expressions of doubt (such as “claims,” “supposedly,” and “insists”), disapproval (such as “resistant” and “non‐compliant”), and racial or class‐based stereotypes.^4^ Such language appears at higher‐than‐average rates in the charts of patients from marginalized groups, especially those of Black patients.^5^ These patterns of bias are not merely semantic. When resident physicians and medical students were asked to read two sample notes documenting a fictionalized patient encounter for sickle cell pain crisis, one containing stigmatizing language and the other more neutral language, those who read the note with stigmatizing language had significantly more negative attitudes toward the patient and were less likely to prescribe pain medication.^6^ This suggests that negative documentation not only alienates patients but also plays a crucial role in compounding, amplifying, and propagating injustice.

In this paper, we argue that undergraduate medical education offers a crucial and largely overlooked avenue for disrupting biased and stigmatizing documentation practices. In 2018, the Council on Medical Education filed a report flagging medical students’ lack of proficiency in clinical documentation, including in both technical and ethical skills, and calling for increased education on the subject.^7^ While some have investigated interventions to improve accuracy and technical proficiency in charting,^8^ there is significantly less literature on attending to the ethical and social consequences of documentation.^9^ The literature that does exist emphasizes that learning how to create clinical documentation is not simply a menial task but also one that structures clinical reasoning and beliefs about patients.^10^ For this reason, we believe that any attempts to improve education on documentation must address ethical skills as well as technical ones. With medical education increasingly recognizing the value of ethics curricula in the formation of competent physicians, discussions of biased language offer one avenue to foreground justice. We contend that incorporating activities designed to elicit critical thinking about language, power, and structural injustice, like those we propose below, can encourage students to reflect upon and improve their own documentation practices and can arm them with a critical lens through which to view the documentation generated by their peers and preceptors as they progress through training and establish the habits that will persist throughout their careers.

## Medical Education as a Point of Intervention

Since the United States Medical Licensing Examination Step 2 Clinical Skills exam was discontinued in 2020, no standardized evaluation assesses the quality of undergraduate medical students’ clinical skills, including documentation,^11^ resulting in little consistency in evaluation and instruction on this topic between medical schools.^12^ The lack of formal education on appropriate language use in documentation results in students’ learning via absorption of observed norms through the “hidden curriculum.”^13^ Medical training is highly hierarchical, and this structure may result in students’ adoption of certain language, phrases, and attitudes held by their senior team members and preceptors due to pressures to assimilate, whether consciously or unconsciously, into the professional norms they witness. For example, medical students who witnessed disparaging humor directed toward patients by the residents and attendings they worked with were more likely to make similar disparaging remarks toward other patients than medical students who did not witness this behavior.^14^ Given that most of the studies documenting negative language in the EHR were conducted at large academic medical centers,^15^ we can assume that this informal curriculum for medical trainees likely includes exposure to patterns of bias and disbelief toward patients. While medical students may face positional obstacles to correcting the behaviors of their senior preceptors if they identify patterns of bias in documentation, the types of exercises we propose below can, at minimum, offer them a framework for thinking about this type of language.

We hope that the exercises we propose will not only enable students to craft stronger and more ethical documentation but also offer one means of interrupting the development of negative attitudes toward patients that sometimes deepen throughout medical education. Concerningly, for example, one of the findings of the sickle cell vignette study was that residents’ responses to the patient were significantly more negative than those of medical students regardless of whether the participants read the stigmatizing or nonstigmatizing chart (though the stigmatizing note heightened this effect).^16^ A recent systematic review of qualitative studies with medical students investigated why empathy declines across medical education.^17^ Some explanations, including overwork, stressful labor conditions, a lack of preparation for clinical work, and exposure to increasingly complex patients, would require more significant changes to the structures of medical education and practice than we offer here. However, several of the factors these students identified, including an emphasis on biomedical knowledge to the exclusion of other skills and the prevalence of negative role models, may be ones that can be addressed by the kinds of exercises we propose. For example, critical instruction on documentation might offer one opportunity for explicit discussion of the communicative and social dynamics of clinical practice. Moreover, we hope that the exercises offered below, by offering students a critical lens through which to view negative role models, will offset the pressure to conform to them.^18^ For these reasons, we encourage incorporating educational content on the nontechnical aspects of documentation as soon as students are exposed to charting practices, and certainly before they begin significant clinical work: if part of trainees’ experience rotating through diverse clinical spaces is to identify skills and attitudes that they hope to emulate or reject in their own practice, priming them to think critically about such language offers grounds for reflection that can be acted on in the future.

## Proposed Interventions: Overall Goals

Our proposed interventions have two major interlocking goals. The first is to encourage trainees to think critically about medical language, from the level of vocabulary to the level of narrative. The assumption that clinical language is neutral and value free is embedded even in the name of the dominant note format: the SOAP (subjective, objective, assessment, plan) note, which juxtaposes the patient's subjective account with the clinician's objective one.^19^ Moreover, as students learn to emulate the highly structured and standardized genre, language that may at first feel foreign or unfamiliar becomes second nature. Thus, our primary aim is to incorporate exercises that defamiliarize this language, prompting students to reflect on the assumptions that underlie common word choices and the implications of choosing one word over another or one explanatory narrative over another. Building directly on this first goal, our second aim is to help students see how these questions of language are directly related to questions of justice and power. Appealing to the growing literature on the unequal distribution of stigmatizing language in medical documentation, we hope to promote health justice by helping students develop a critical consciousness that allows them to see how the language of medicine can reflect bias and perpetuate injustice.

Below, we make a series of suggestions for methods through which the insights of the scholarly literature on biased documentation can be incorporated into medical education in support of these goals. Wherever possible, we encourage educators to draw on the material generated by recent studies rather than reinventing the wheel. We believe that the exercises below would be deployed most fruitfully at the time that students are first being introduced to the technical skills involved in clinical documentation so that these themes are front of mind as they begin to form long‐standing habits. While we imagine these activities taking place in a venue like an hour‐long communication skills or ethics workshop, we offer these suggestions as starting points that instructors should adapt and develop in ways that best suit their institutional context. Ultimately, we argue, offering preclinical students explicit education on the ethics of medical language provides a critical lens that they can deploy as they develop their professional habits and identities and deepen introspection on the power of the language they will wield as medical professionals.

## Exercise 1: Thinking Critically about Language and Power through Comparison Cases

Clinical language is not merely neutral; it is a powerful force for structuring both individual thought and cultural beliefs about health.^20^ Narrative medicine has thus brought the tools of literary analysis to bear on medical language, revealing the underlying assumptions and nuances of linguistic choices.^21^ The first exercise that we suggest brings together the insights of such analysis with scholarship on stigmatizing language in charts.

Facilitators should begin by asking students to compare the language of two sample charts. We recommend that instructors use (with citation) the sample notes generated by Anna P. Goddu et al., as the language was abstracted from trends they observed in charts at their own institution. After giving students time to read through the two notes, preceptors should ask them to identify as many differences as possible, encouraging them to pick up on minor details, like noting that a patient comes in “with 10/10 pain” versus “stating he has 10/10 pain.”^22^ Once the students have generated a robust list, they can work through it by describing the connotations, rather than denotations, of common phrases in the EHR. For example, the first note frames the patient's pain as a fact, and the second as a subjective and even suspicious account. By offering alternatives to language that may initially register as “neutral” or “objective” to students, this relatively simple exercise can help defamiliarize commonly used language and reveal the underlying implications baked into the generic conventions students are being taught.

In addition to allowing students to explore the power of seemingly minor linguistic differences, comparing the two charts offers an opportunity to address more explicitly discriminatory attitudes. Many of the differences between the two are intended to embed signals of race and class into the chart, as in quoting a patient as saying that he has pain “all up in my arms and legs.”^23^ When students raise concerns about the use of this kind of language, they should be prompted to explore it more deeply: Why do they think someone would include language of this kind in a chart? What is it intended to communicate and to whom? What do they think the patient or a family member would think if they read the chart? What concrete implications might it have for the patient's future care? By practicing the identification and analysis of stigmatized language, students can be encouraged to consider the bias in, audience for, and impact of such language.

After students have had the opportunity to make their own observations and analyses, we encourage facilitators to introduce material from the scholarly literature. For instance, they might introduce a list of themes from the literature on types of stigmatizing language, as well as the literature on their disproportionate use regarding members of marginalized groups, as cited in our introductory material. For example, resources such as a recent scoping review by Megan Healy et al. offer both specific terms to avoid (including “druggie,” “frequent flyer,” and “promiscuous”) as well as more general strategies: “(1) use person‐first language, (2) eliminate pejorative terms, (3) make communication inclusive, (4) avoid labels, (5) stop weaponizing quotes, (6) avoid blaming patients, and (7) abandon the practice of leading with social identifiers.”^24^ The authors offer concrete examples to illustrate each theme, allowing for easy integration of actionable suggestions into educational material.

In addition to providing resources for creating more ethical and patient‐centered documentation, instructors should clarify the concrete impacts of linguistic choices. The clearest way to do this is by revealing the insights of Goddu et al.'s experiment on how the chart language impacted trainees’ attitudes toward the fictional patient and comfort in offering pain medication. Further examples could drive the point home: For instance, in one study, mental health professionals randomly assigned vignettes in which a patient is described either as a “substance abuser” or as “having a substance use disorder,” and participants were more likely to endorse individual culpability and punitive response when they read the former term.^25^ By introducing this literature after students’ active engagement with these themes, instructors can validate and expand upon students’ observations in support of our twin goals of heightened critical thinking about language and power. Thus, we intend for this exercise not only to reduce biased language usage in a new generation of clinicians but also to deepen students’ thinking about the power they hold as the official documenters of patients’ stories.

## Exercise 2: Thinking about Perspective, Humility, and Structure

One of the most common themes in the literature on negative language in documentation is the use of language expressing paternalistic attitudes toward patients, such as “I have instructed her” and “I impressed upon her the importance of …”^26^ This type of language may be particularly indicative of racial bias. Negative language implying difficulty, like “resistant” or “noncompliant,” appears over 2.5 times more often in the charts of Black patients than white ones.^27^ The language of compliance, we suggest, creates a narrative in which it appears that the patient rejects sound medical advice for inconsequential or antagonistic reasons. Our second proposed activity encourages students to deepen their examination of language and power through a perspective‐taking exercise that asks them to expand their imagination through a consideration of such compliance narratives. It expands on our goals in the first exercise in two key ways: first, by turning inquiry from the level of the word or phrase to the level of the narrative and, second, by moving from a consideration of language as a marker and communicator of individual attitudes to one that emphasizes structural injustice. Ideally, this session would occur several weeks to months later in a longitudinal clinical skills curriculum to offer the ability to revisit and reinforce prior learning.

For this exercise, students should be shown a hypothetical clinical note describing a difficult patient encounter in which a “noncompliant” patient disagrees with a clinician's recommended treatment. For example, the note might include language like “the patient declines admission despite ongoing demonstration of increased oxygen need from baseline because he's ‘got rights and ain't got time for hospitals.’ He is counseled extensively about the risks of declining hospitalization, especially given that he has been repeatedly noncompliant regarding medications in the past.” Students can first be led through the same kinds of questions introduced in our first exercise: What do words like “noncompliant” or the use of “extensively” as an intensifier of “counseled” signal about whose priorities should guide medical decision‐making? What do they understand as the purpose and motivation of “compliance talk”? What alternatives do they see to this kind of language? How might such language challenge patient autonomy? Or justice? What might the concrete impacts of this language be on patients’ subsequent care?

After reactivating students’ thinking about the impact of clinical language, preceptors can ask students to spend ten minutes freewriting about how they imagine the encounter from the patient's point of view. Perspective‐taking exercises have been encouraged in educational literature for their ability to encourage critical thinking, protect against burnout, and improve student wellness. One curriculum for teaching students to write patient‐centered documentation^28^ asks students to imagine patient perspectives; however, this exercise, framed in the context of open notes legislation, asks students to read the note from the patient's point of view.^29^ While we agree that this is a worthwhile exercise, our proposal takes a further step, asking students to renarrate the encounter as a whole, imagining what might be missed when assumptions are made about the motivations for rejecting a clinician's recommendations, and reminding them that the clinician's point of view is only one perspective within the patient's larger story. Thus, while the official medical chart might narrate resistance or foolishness, students might imagine pressing family‐care obligations, fears of impossible hospital bills, or past medical trauma as contributors to the patient's decisions. After writing, students can be prompted to share their accounts, explaining why they opted for particular narratives.

We hope that this reminder of the myriad motivations, contexts, or personal histories that might shape a patient's interactions with health care providers paradoxically strengthens students’ development of “narrative humility,” which recognizes that “our patients’ stories are not objects that we can comprehend or master but, rather, dynamic entities that we can approach and engage with while simultaneously remaining open to their ambiguity and contradiction.”^30^ Cultivating narrative humility provides students with a greater awareness of their own values, prejudices, and “frames of listening” and how these color their perceptions of the patient. Students should thus be encouraged to reflect upon their assumptions about patient reasoning and to question the reflex to frame differences about medical decisions as stories about “noncompliance” in which the clinician is the default protagonist.

Narrative humility is particularly important given the relative power of physician‐authored and patient‐authored stories. Within medicine, the physician‐authored narrative in the patient's chart becomes the authoritative one. We thus also intend that this exercise offer an opportunity to strengthen students’ structural competency. Jonathan Metzl and Helena Hansen introduced the concept of *structural competency* to suggest that education surrounding bias and stigma should move beyond a purely interpersonal focus to center instead on the socioeconomic, legal, historical, and institutional drivers of health. Structural competency offers students the language and tools to observe and describe these forces that exist beyond the clinic and challenges students to consider interventions at these higher levels.^31^ Educators have fruitfully incorporated initiatives designed to advance structural competency across health professions programs.^32^ When illustrating the concept, for example, Metzl and Hansen propose that students might be offered a short vignette: “Mrs. Jones is an African American woman in her mid‐60s who comes late to her office visit and refuses to take her blood pressure medication as prescribed.” Rather than asking for the kind of analysis typical in a cultural competency approach, which might ask about her personal or cultural beliefs about medications, they suggest that students analyze this vignette through the lens of structural factors beyond the clinical encounter, such as the time pressure of the exam due to insurance or institutional policies, the policies dictating the price of medications, or access to pharmacies and grocery stores in her neighborhood.

To help students think structurally during this exercise, facilitators can pick up on and encourage the further discussion of the kinds of upstream concerns that will emerge in many students’ renarrated encounters: Why has the patient described in our vignette been “noncompliant” with medication in the past? Why does the patient feel that they do not have time for a hospital admission? By drawing attention to students’ explanatory models, preceptors can foreground the question of personal responsibility, discussing how terms like “noncompliance” assign blameworthiness to patient decision‐making. Biased language both reflects and reinforces ideologies that view patients as personally responsible for their health outcomes, rather than as part of a larger sociopolitical context. Emphasizing that medical decisions are often the product of structural factors can also strengthen students’ understanding that language implying blame not only stigmatizes a specific individual but can also disguise deeper issues that might otherwise be uncovered and mitigated.

Finally, students can be prompted to think about the chart itself as an administrative structure by considering how the language they document within the patient's medical record travels with the patient, shaping the kind of care they will receive in subsequent visits. They can be asked, for example, to consider how their choice of terminology might impact the person's future interactions with health care professionals or the criminal justice system. In this way, discussing how the power and permanence of the EHR form one of many structures that shape patients’ future health care outcomes can help students become more structurally competent in the domain of medical documentation and beyond.

## Finding the Words: Language as a Source of Insight

The use of stigmatizing language in medical documentation is insufficiently addressed in medical education and serves as a barrier to achieving patient‐centered care and health justice. We propose that medical education can intervene in this issue by challenging students first to think critically about medical language in documentation and then to analyze how this language relates to deeper power structures and justice.

While we have focused on instruction that should be incorporated into students’ earliest exposure to documentation, we anticipate that lessons derived from these materials and exercises will require maintenance. Notably, students who took part in a workshop on writing notes in the context of open notes legislation indicated that they had gained confidence in generating patient‐centered documentation; however, several of them identified the disjuncture between the practices they were taught and the norms and expectations of their clinical preceptors.^33^ Thus, we encourage medical educators to consider how they could revisit these topics and exercises after students enter clinical rotations to allow reflection on how these experiences have changed their perspectives. Candid discussions about their experience navigating this hidden curriculum could offer students the opportunity to review their education on the power of clinical language and reaffirm their commitment to evolving best practices.

The success of these measures is not easy to assess. One possible mechanism for evaluating students’ integration of such content is the evaluation of notes that they generate in coursework and beyond: Kathleen Eng et al. developed a rubric for student notes that incorporates a category on the use of patient‐centered language, offering one way to evaluate students’ ability to integrate the material taught in such sessions. However, while our most obvious and tangible goal is to shift documentation practices in a new generation of clinicians, we also see the activities and discussions outlined above as opportunities to engage students in thinking more deeply about the power their language will hold as they assume clinical roles. Thus, we recommend that the success of these interventions can best be evaluated through qualitative analysis of student and faculty reflections on the exercises to determine if they have elicited the kind of critical thinking that we seek to instill.

We believe that education can shift clinicians’ understanding of the language they use and help to alter practices in a new generation and that these changes can directly contribute to the provision of more patient‐centered and less‐biased care. Medical charts occupy a unique space between the individual and the institutional: while capturing encounters with specific patients, they rely on highly patterned generic norms that are propagated through formal and informal education and through highly structured digital interfaces; written by individual clinicians following discrete encounters, they become part of a medical and bureaucratic apparatus that travels with a patient for years via the EHR. Empowering medical students to think critically about the language of clinical documentation, we suggest, offers opportunities to interrupt the perpetuation of stigmatized rhetoric from the microlevel (as in the case of implicit and explicit bias on the part of the provider) to the macrolevel (such as the modeling of stigmatizing rhetoric by supervisors in medical training, the availability and use of templates and copy‐and‐paste functions within the EHR, or working conditions that contribute to burnout or hastily written notes). Ultimately, although negative language may in part be only a symptom of larger structural issues within health care and society at large, we can still offer students a critical lens through which to view and shape the documentation they will read and generate as they enter the clinic.
